# Familial Transmission of *emm12* Group A *Streptococcus*

**DOI:** 10.3201/eid2310.170343

**Published:** 2017-10

**Authors:** Claire Duployez, Anne Vachée, Olivier Robineau, François Giraud, Anthony Deny, Eric Senneville, Frédéric Wallet, Caroline Loïez

**Affiliations:** Centre Hospitalier de Roubaix, Roubaix, France (C. Duployez, A. Vachée, F. Giraud);; Centre Hospitalier Régional Universitaire de Lille, Lille, France (C. Duployez, A. Deny, F. Wallet, C. Loïez);; Centre Hospitalier de Tourcoing, Tourcoing, France (O. Robineau, E. Senneville);; University of Lille, Lille (O. Robineau, E. Senneville);; Centre de Référence des Infections Ostéo-Articulaires Complexes Nord-Ouest, Tourcoing (E. Senneville)

**Keywords:** Streptococcus pyogenes, group A streptococcal infections, iGAS, emm12, familial, invasive, leukocyte, polymorphonuclear, C-reactive, procalcitonin, nonsteroidal anti-inflammatory drugs, NSAIDs, antimicrobial drug, antibiotic, antibacterial, amoxicillin-clavulanic acid, linezolid, group A Streptococcus, bacteria

## Abstract

Incidence and severity of invasive group A *Streptococcus* infections are of increasing concern in France and worldwide. The risk for secondary infection of close contacts is known but rarely described. We report a case of intrafamilial and life-threatening transmission of *emm12* group A *Streptococcus*.

In recent years, incidence and severity of invasive group A *Streptococcus* infections (iGAS) has increased in Europe and worldwide (*1*). The Centers for Disease Control and Prevention surveillance reports attest to this increase in the United States (*2*). In northern France, an increase in *emm1* iGAS was reported (*3*). *Streptococcus pyogenes* can spread from infected persons to close contacts, especially if >24 hours is spent with an infected person (*4*). However, transmission of life-threatening infections remains a relatively rare event. We report a case of intrafamilial transmission of iGAS.

On August 17, 2016, a 67-year-old woman was admitted to the Centre Hospitalier de Roubaix (Roubaix, France) for knee pain and necrotic zones on her thigh. Her medical history consisted of treated hypertension. At admission, her temperature was 36.3°C, blood pressure 110/80 mm Hg, pulse rate 74 beats/min, blood leukocyte count 8,530 cells/µL (89.9% polymorphonuclear), C-reactive protein 281 mg/L, and procalcitonin 32.5 ng/mL. She had acute renal failure (creatinine 35 mg/L) and abnormal clotting test results. We collected a set of blood specimens for culture. She had a recent history of erysipelas and was given nonsteroidal antiinflammatory drugs (NSAIDs) the day before her hospital admission. We diagnosed necrotizing fasciitis of the leg. She received intravenous antimicrobial drug therapy with amoxicillin/clavulanic acid, gentamicin, and clindamycin and underwent debridement surgery on the same day. 

Her health condition quickly deteriorated; she had disseminated intravascular coagulation and blood pressure of 70/40 mm Hg, despite appropriate hemodynamic care. She experienced toxic shock and multiorgan system failure. A revision surgery was necessary but not possible because of heavy bleeding, hemodynamic instability, metabolic acidosis, acute renal failure, and hyperkaliemia. She died on August 18. Cultures of necrotized tissues and blood samples yielded *S. pyogenes*. 

On August 21, the index case-patient’s husband, who was 66 years of age, was admitted to Centre Hospitalier Régional Universitaire de Lille (Lille, France) with a 2-day history of bursitis of the right elbow. He had been treated during the 2 days by his family’s physician with intravenous amoxicillin/clavulanic acid (1 g 3×/d), pristinamycin (1 g 3×/d), and NSAIDs. At hospital admission, his temperature was 36.5°C, blood pressure 95/55 mm Hg, and pulse rate 95 beats/min, blood leukocyte count 22,300 cells/µL (89% polymorphonuclear), and C-reactive protein 511 mg/L. Mobilization of the elbow was possible but limited by major edema to the axilla; severe blistering of the elbow was visible. We drained his forearm surgically to treat extensive cellulitis and diagnosed superinfected bursitis. We stopped pristinamycin and NSAIDs, increased the intravenous amoxicillin/clavulanic acid dose (to 2 g 3×/d), added linezolid for antitoxinic action (600 mg 2× in 24 h), and provided 14 hyperbaric oxygen therapy sessions. On August 23, the wound condition improved and C-reactive protein decreased (211 mg/L), but a wide erythema was still visible. Clinical outcome was favorable, and we discharged the patient on September 5. Culture of the deep tissues samples yielded *S. pyogenes*. The strain was susceptible to amoxicillin, so we continued it (2 g 3×/d) until September 11.

Strains isolated from both patients were the same strain of *S. pyogenes emm*12 (Technical Appendix Tables 1, 2). The couple were caregivers for their granddaughter, and they met their 2 adult children and son-in-law several days each week, so we evaluated these close contacts. The son and daughter had had a sore throat 9 days before onset of illness in the mother and were treated empirically by their general practitioner with amoxicillin/clavulanic acid (1 g 2×/d for 6 d). We prescribed cefuroxime axetil (250 mg 2×/d for 10 d). Buccal swabs cultured remained negative.

The risk for iGAS infection in close contacts of patients was reviewed in 2016 (*5*): the evidence was based on 13 instances of transmission published in 4 separate studies covering 5,858 household contacts. The annual risk among close contacts was 151 times greater than the risk for sporadic disease and comparable to that estimated for meningococcal disease. However, the benefit from antimicrobial drug prophylaxis is not known (*5*), and guidelines vary among countries. In the United Kingdom, prophylaxis is recommended for exposed mothers or babies during the neonatal period, for symptomatic close contacts, or for the entire household if there is >1 case (*6*). In Canada, prophylaxis is recommended for persons who had close contact with a person with a confirmed severe case during a specified period (*7*); in France and the United States, prophylaxis is recommended for close contacts with risk factors for invasive infections (*8**,**9*). In the cases we report here, the second case-patient did not receive prophylaxis because of the short period between the 2 cases. 

Both case-patients received NSAIDs during the onset of the disease. The role of these drugs in streptococcal infection outcome is frequently discussed; they seem to cause an increase of severe infection, most probably in children (*10**).*

These cases highlight that different life-threatening transmissible types of *S. pyogenes* are circulating in the same area and that transmission can occur rapidly. Clinician and family education about prophylaxis and symptoms requiring medical care is needed.

Technical Appendix Antimicrobial susceptibility test results and strain analysis of *emm*12 group A *Streptococcus* *pyogenes* spread by familial transmission, France.
