# Supplementary material for: Familial Transmission of emm12 Group A Streptococcus
Source: Emerg Infect Dis. 2017 Oct;23(10):1745–6. doi: 10.3201/eid2310.170343 (PMC5621563; doi:10.3201/eid2310.170343)
Supplement: Technical Appendix — Antimicrobial susceptibility test results and strain analysis of emm12 group A Streptococcus pyogenes spread by familial transmission, France. [file 17-0343-Techapp-s1.pdf]

Article DOI: https://doi.org/10.3201/eid2310.170343

Familial Transmission of *emm12* Group A *Streptococcus*

Technical Appendix

Table 1. Antimicrobial susceptibility test results of *emm*12 Group A *Streptococcus pyogenes* spread by familial transmission*†

	Antimicrobial susceptibility testing (disk diffusion method)‡	
Antimicrobial drug	Case-patient 1	Case-patient 2
Penicillin G	S	S
Ampicillin	S	S
Amoxicillin	S	S
Cefotaxime	S	S
Gentamicin	I	I
Tetracycline	S	S
Levofloxacin	S	S
Erythromycin	S	S
Lincomycin	S	S
Pristinamycin	S	S
Linezolid	S	S
Teicoplanin	S	S
Vancomycin	S	S
Nitrofurantoin	S	S
Rifampin	S	S

*S, susceptible; I, intermediate categorization.
†The 2 strains of *Streptococcus pyogenes* were identified by the hospital laboratories and by the Centre National de Référence des Streptocoques using mass spectrometry.
‡Antimicrobial susceptibility testing and genotyping of the 2 strains were performed simultaneously by the Centre National de Référence des Streptocoques.

Table 2. Strain analysis of *emm*12 Group A *Streptococcus pyogenes* spread by familial transmission

	Toxins and superantigens genotyping	
	Case-patient 1	Case-patient 2
Specimen*	*emm*12	*emm*12
*Spe*A	Negative	Negative
*Spe*B	Positive	Positive
*Spe*C	Positive	Positive
*Ss*A	Negative	Negative
Sic	Negative	Negative
Smez	Positive	Positive

*Protein M genotyping (sequencing of the N terminus variable region of the ***emm*** gene).
